# Development and validation of an instrument for measuring competencies on public health informatics of primary health care worker (PHIC4PHC) in Indonesia

**DOI:** 10.1017/S1463423620000018

**Published:** 2020-07-06

**Authors:** Enny Rachmani, Chien-Yeh Hsu, Peter WuShou Chang, Anis Fuad, Nurjanah Nurjanah, Guruh Fajar Shidik, Dina Nur Anggraini Ningrum, Ming-Chin Lin

**Affiliations:** 1 College of Medical Science and Technology, Graduate Institute of Biomedical Informatics, Taipei Medical University, Taipei City, Taiwan; 2 Department of Health Information Management, Faculty of Health Science, Universitas Dian Nuswantoro, Semarang, Jawa Tengah, Indonesia; 3 Department of Information Management, National Taipei University of Nursing and Health Science, Taipei City, Taiwan; 4 Master Program in Global Health and Development, Taipei Medical University, Taipei City, Taiwan; 5 TUFTS University Medical School, Chung Shan Medical University, Taichung, Taiwan; 6 Department of Biostatistics, Epidemiology and Population Health, Public Health, Faculty of Medicine, Gajah Mada University, Yogjakarta, Indonesia; 7 Department of Public Health, Faculty of Health Science, Universitas Dian Nuswantoro, Kota Semarang, Jawa Tengah, Indonesia; 8 Department of Informatics, Faculty of Computer Science, Universitas Dian Nuswantoro, Semarang, Jawa Tengah, Indonesia; 9 Department of Public Health, Semarang State University, Kota Semarang, Jawa Tengah, Indonesia; 10 Department of Surgery, Division of Neurosurgery, Taipei Medical University-Shuang Ho Hospital, New Taipei City, Taiwan; 11 International Center for Health Information Technology (ICHIT), Taipei Medical University, Taipei, Taiwan

**Keywords:** computer literacy, developing countries, health information system, primary health care, public health informatics

## Abstract

Because of the increasing adoption and use of technology in primary health care (PHC), public health informatics competencies (PHIC) are becoming essential for public health workers. Unfortunately, no studies have measured PHIC in resource-limited setting. This paper describes the process of developing and validating Public Health Informatics Competencies for Primary Health Care (PHIC4PHC), an instrument for measuring PHC workers’ competencies in public health informatics. Method: This study developed a questionnaire that had three stages: the Delphi technique, a pretest, and field test. Eleven academicians from a university and 13 PHC workers joined 2 rounds of group discussion in the first stage. The second stage comprised two pilot studies with 75 PHC workers in Semarang Municipality. The third stage involved validating the questionnaire with 462 PHC workers in Kendal District. This study used Pearson’s product-moment correlation for the validity check and Cronbach’s alpha coefficient for determining the internal consistency. This study used the K-means algorithm for clustering the results of the PHIC4PHC questionnaire. Results and Conclusion: PHIC4PHC is the first comprehensive PHIC questionnaire administered in a resource-limited setting, consisting of 11 indicators and 42 measurement items concerning knowledge of health information systems, skills required for health data management, ethical aspects of data sharing and health information literacy. The final results of PHIC4PHC were clustered into three classes based on the K-means algorithm. Overall, 45.7% PHC workers achieved medium competency, whereas 25.6% and 27.7% achieved low and high competency, respectively. Men had higher competency than women. The higher the worker’s level of education, the higher the PHIC level; the longer the worker’s work experience, the lower the PHIC score; and the greater the worker’s age, the lower the PHIC score. Measuring and monitoring PHIC is vital to support successful health IT adoption in PHC.

## Introduction

The resolution of universal health coverage (UHC) by the UN General Assembly in December 2012 identified UHC as a central global health objective (Vega, 2013). This challenge should place health workers at the center of each country’s response, including its stock, skill mix, distribution, productivity, and quality. Particularly, in the context of low- and middle-income countries, gaining competent health workers is a foundation for accelerating the attainment of UHC (Campbell *et al*., 2013).

In many low- and middle-income countries, primary health care (PHC) has been chosen as the primary strategy to achieve equitable, patient- and community-oriented and comprehensive approaches to achieving UHC (Sachs, 2012). Developing the function of PHC is crucial to serving people in remote areas where PHC requires improvements in infrastructure, skilled human resources in health care, appropriate health technologies, and financial support, as well as comprehensive health program management (Hall and Taylor, 2003; De Maeseneer *et al*., 2007; WHO, 2014).

Studies have found that improving communication networks and internet availability have improved access to health information and furthermore could elevate digital health literacy in the community, including among PHC workers (Edejer, 2000; Berland *et al*., 2001; Cline and Haynes, 2001; Norman and Skinner, 2006b; Gilmour, 2007; Bujnowska-Fedak, 2015; Vâjâean and Bãban, 2015). Several studies have reported the increasing use of health information technologies in various programs in PHC, such as inpatient electronic registry, processing, and evaluation programs and management, clinical decision support systems, surveillance, and patients monitoring (Pambudi *et al*., 2004; Tomasi *et al*., 2004; Ludwick and Doucette, 2009; Tomasi *et al*., 2009; Denomme *et al*., 2011; Rachmani *et al*., 2013; Yazdi-Feyzabadi *et al*., 2015). Some countries still report poor performance despite having dedicated information systems for PHC (Belanger *et al*., 2012; Farahat *et al*., 2018). Different settings pose specific challenges during implementation, and human resources have been considered as the most critical among the factors that contribute to the success of health IT in PHC (Ludwick and Doucette, 2009).

In the 21st century, public health professionals face major challenges, particularly in terms of technological advances, and demographic changes (Hernandez *et al*., 2003). Public health informatics competencies (PHIC) have become critical to PHC workers because of the current trend of health IT adoption and its necessity for jobs in PHC to be performed efficiently (Alpay *et al*., 2000; Montague, 2014). Public health informatics (PHI) is the application of computer science and information technology systems to public health practice, research, and learning (Friede *et al*., 1995). It integrates public health and IT and consists of four knowledge domains; organization and management systems, public health, information system and IT (Magnuson and Fu, 2014). PHI could improve public health surveillance capacity and response, but confidentiality and security of the information systems is a concern (Hernandez *et al*., 2003).

Public health workers should be able to support public health decisions by facilitating the availability of timely, relevant, and high-quality information. In other words, they should always be able to provide advice on methods for achieving a public health goal faster, better, or at a lower cost by leveraging computer science, information science, or technology (Savel and Foldy, 2012). Furthermore, public health professionals in PHC need to understand many facets of health care, including public health, health promotion, health services research, and information and communication technology (Joshi and Perin, 2012).

Indonesia is a developing country committed to achieving UHC by 2019 (Simmonds and Hort, 2013). Indonesia has 9859 PHC facilities distributed across the archipelago serving an estimated 250 million people. In 2011, among all PHC facilities, 78.4% had computers and 46% had adopted PHC information systems. (Indonesia, 2011; Indonesia, 2016). The number of PHC facilities with a health information system (HIS) has increased dramatically because of the enactment of a national social security program in 2014. In this regard, measuring the PHIC of PHC workers is crucial to ensure optimal functioning of PHC activities.

A recent study described PHIC in developed countries and for the mid-tier level of health professionals (Hsu *et al*., 2012). As of yet, no studies have measured the PHIC of PHC workers in low- and middle-income countries despite their adoption of technology into PHC. The objective of this study was to develop an assessment instrument to measure PHIC in the domain of information systems and IT for PHC workers with limited education and resources in developing countries. The instrument was developed in three stages: first, constructing categories, indicators, and items for the questionnaire; second, conducting a pretest via two pilot tests; and third, conducting a field test with PHC workers. This study used the Delphi technique to construct the questionnaire items with the judgment of experts to generate the final set of questionnaire items.

## Methods

### Research setting and design

**Figure 1.**
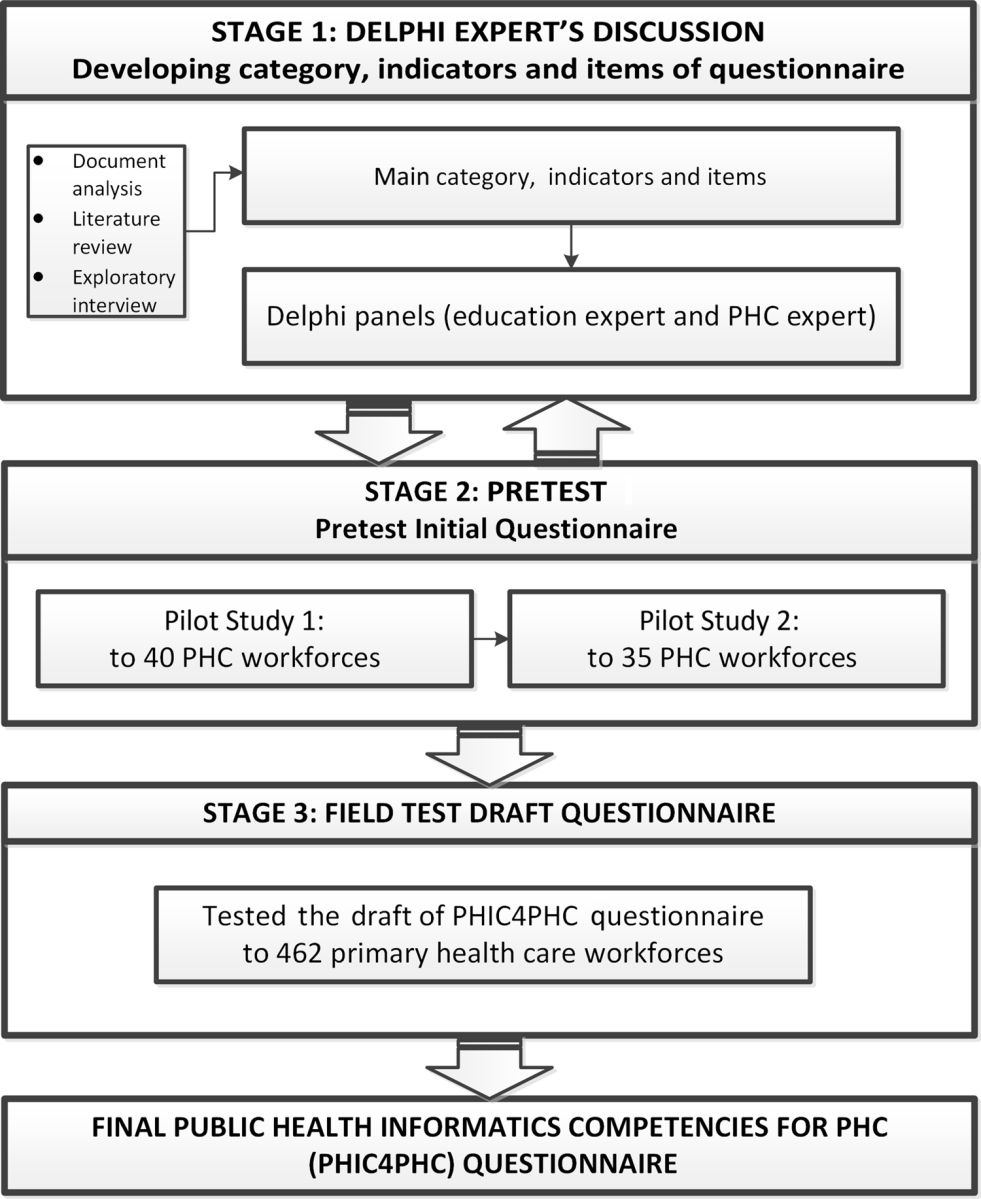
Steps in developing and validating a PHIC4PHC instrument

This study had three stages (Figure 1): the first stage was development of the instrument, the second stage was pretest studies, and the third stage was field testing of the questionnaire. The first and second stages were conducted in Semarang Municipality, Central Java Province of Indonesia. The third stage was conducted in Kendal District, Central Java Province of Indonesia in 25 PHC facilities. Semarang Municipality was used to obtain experts opinion and conduct the pretest because it is an urban city in which people are more exposed to technology, whereas Kendal District is a rural-urban city and can therefore represent the characteristic of Indonesia’s PHC facilities, which are located in both rural and urban area.

This study used the Delphi technique to challenge and construct the Public Health Informatics Competencies for Primary Health Care (PHIC4PHC) questionnaire. The Delphi technique is a method for structuring group communication using a series of questionnaires. The technique can ensure that the communication process is effective and that a consensus can be reached between the researcher and a group of experts on a specified topic. The technique is used when the opinions and judgment of experts are needed but precise information is unavailable (Hasson *et al*., 2000; Hsu and Sandford, 2007; Keller and Heiko, 2014). This study employed a modified Delphi through two steps with face-to-face meetings of experts on education and PHC. The purpose of the meetings was to achieve a consensus regarding the construction of the questionnaire. Furthermore, the expert opinions were also used in the second stage to reduce the numbers of questionnaire items. The purposive snowball sampling technique was used to identify experts who know other experts with similar characteristics such as knowledge, skills, and experiences (Biernacki and Waldorf, 1981; Palinkas *et al*., 2015). Experts including academicians and PHC workers evaluated a list of potential competencies derived from a literature review. Eleven academicians participated in the first round and 13 PHC practitioners joined the second round.

An instrument agreed upon in the first stage was pretested in two pilot studies in the second stage. In the first pretest, 40 public health workers filled in the questionnaire at a Gunung Pati PHC facility, and the following pretest involved 35 public health workers at Semarang Municipality Health Office.

Figure 1 shows the flow of this study and the three-stage construction of the PHI4PHC questionnaire. The participants in the first and the second stages were 24 experts and 75 public health workers, respectively. The third stage involved 462 PHC workers filling out the PHIC4PHC questionnaire in the field test.

### Stage 1: instrument development

#### Literature review

A literature review was performed to assess recent instruments acknowledged as information and communication technology (ICT) measurement tools to obtain a comprehensive viewpoint for developing PHIC assessment tools. Previous studies have developed instruments for measuring computer literacy, computer competency, computer knowledge, computer usage, attitudes toward computers, ICT literacy, and so forth but few have specialized in health. Table 1 provides a brief description of 10 computer literacy and competency measurement tools. The table shows that the computer-email-web fluency and eHealth Literacy Scale (eHeals) tools included the internet as a component of digital technology in their constructs; however, only one instrument is concerned with health.

**Table 1.** Recent instruments for measuring ICT literacy

Name	Description	Items	Source
Windows Computer Experience Questionnaire	The questionnaire is a comparatively short measurement instrument.	13 items	(Miller *et al*., 1997)
Computer Understanding and Experience Scale	The instrument is a self-report measure of computer experience.	12 items	(Potosky and Bobko, 1998)
Subjective Computer Experience Scale	The instrument is used to assess behavioral beliefs, outcome evaluation, and global attitude toward email, using three subscales.	62 items	(Smith *et al*., 2007)
Computer Use Scale	The scale measures four dimensions of the different ways in which people use computers.	62 items	(Panero *et al*., 1997)
Computer Ability Survey	The survey assesses and predicts an adult learner’s ability to use computers.	22 items.	(Kay, 1993)
ETS iCritical Thinking (formerly *iSkills*, formerly *ICT Literacy Assessment*) by the International ICT Literacy Panel (2001)	Developed in response to a need for large-scale institutional assessment of information literacy and technical skills grounded in cognitive and problem-solving skills.	60 items	http://www.ets.org (Katz, 2007)
Project SAILS (Standardized Assessment of Information Literacy Skills) for higher education students	An instrument with a large-scale, knowledge-based multiple-choice test, featuring a variety of basic and advanced information literacy skills and concepts.	45 items	https://www.projectsails.org/ (Radcliff *et al*., 2007)
Digital Competence Assessment (iDCA)	This tool covers technological, cognitive, and ethical issues.	85 items	(Calvani *et al*., 2009)
Computer-email-web (CEW) fluency scale	An instrument for assessing people’s fluency with computers, email, and the web.	21 items	(Bunz, 2004)
e-Health Literacy Scale (eHeals)	A tool for measuring consumers’ combined knowledge of, comfort with, and perceived skills in finding and evaluating electronic health information and applying it to health problems	8 items	(Norman and Skinner, 2006a)

The initial structure of PHIC assessment tool in this study was based on a framework of ICT literacy, Digital Competence Assessment, and the eHeals because this study focused on information system and IT competencies specific to the health area (Panel, 2002; Norman and Skinner, 2006a; Cartelli *et al*., 2012). The concept of the PHIC assessment tool in this study consists of technical, ethical, and cognitive competencies and health information literacy.

#### Designing the main categories, indicators, and items

The process for developing the questionnaire items in this study was based on previous studies on ICT literacy in the health area (Jiang *et al*., 2004; Yang *et al*., 2004; Norman and Skinner, 2006a; Neter and Brainin, 2012; Gürdaş Topkaya and Kaya, 2015). As shown in Table 2, the PHIC4PHC has 4 main categories or domains of *cognitive proficiency*, *technical proficiency*, *and ethical proficiency*, and *health information literacy*, in addition to 12 indicators. This step created the initial version of the questionnaire (PHI4PHC v.0) consisting of 85 questions rated on a 5-point Likert scale (where 1 is strongly unimportant and 5 is strongly important) as shown in Table 3.

**Table 2.** Ranking of indicators in the research process

Proficiency domain	Main indicator	Education experts			PHC experts			Pilot Test 1			Pilot Test 2		
		Rank	Mean	Items	Rank	Mean	Items	Rank	Mean	Items	Rank	Mean	Items
Cognitive proficiency	HIS knowledge	8	4.0	11	7	4.5	9	8	3.9	9	5	4.1	9
	HIS skill	9	3.8	8	8	4.5	3	11	3.8	3	8	3.9	3
Technical proficiency	General skill	11	3.8	29	4	4.6	10	6	4.0	12	9	3.9	10
	Office skills	7	4.0	14	10	4.5	9	9	3.9	9	11	3.6	9
	Network skill	12	3.5	8	1	4.8	2	3	4.2	2	7	3.9	2
Ethical proficiency	Legal knowledge	5	4.2	3	11	4.5	1	10	3.8	1	6	4.0	1
	Security knowledge	10	3.8	2	12	0.0	0	12	0.0	0	12	0.0	0
	Privacy knowledge	6	4.1	2	2	4.8	1	1	4.3	1	1	4.3	1
Health information literacy	Access	1	5.0	2	9	4.5	2	2	4.3	2	2	4.2	2
	Manage	2	5.0	2	3	4.7	2	4	4.2	2	3	4.1	2
	Integrate	3	4.6	2	5	4.6	2	5	4.2	2	4	4.1	2
	Evaluate	4	4.5	2	6	4.6	2	7	4.0	2	10	3.8	2
				85			43			46			43

**Table 3.** Reduction of questionnaire items via the experts panel

No	PHI4PHC ver.0	Experts	
		ver.1	ver.2
1	PHC workers should know the recent computer systems such as Windows, Macintosh.	valid (4.0)	valid (4.5)
2	PHC workers should know general computer terminologies, such as RAM, ROM, HD	valid (4)	deleted
3	PHC workers should know basic components of computer hardware and their functions	not valid (3.6)	deleted
4	PHC workers should know the input and output computer devices	not valid (3.6)	deleted
5	PHC workers should know the basic components of computer software and their functions	not valid (3.9)	deleted
6	PHC workers should know the basic usage of a computer, such as shutting down, using of mouse	valid (4.9)	valid (4.9)
7	PHC workers should know the function of the file management system in the system operation, such as create, copy, move the folder or file	valid (4.8)	valid (4.7)
8	PHC workers should know how to use an operating system, such as Windows	valid (4.6)	valid (4.6)
9	PHC workers should know how to install the drivers of computer appliance, such as printer, scanner.	valid (4.0)	valid (4.6)
10	PHC workers should know how to assemble computer devices	not valid (2.4)	deleted
11	PHC workers should be able to solve the general problem of error condition	not valid (3.4)	*Can solve the general problem of error condition (**reinstated**)*
12	PHC workers should know the basic principles of computer network	not valid (3.2)	deleted
13	PHC workers should know the basic structure of computer network	not valid (3.0)	deleted
14	PHC workers should know the type of the network in the workspace	not valid (2.7)	deleted
15	PHC workers should know the type of main computer network that have been used now	not valid(3.0)	deleted
16	PHC workers should know how to set up computer communication software	not valid (2.6)	deleted
17	PHC workers should know the different digital and analog signals	not valid (2.6)	deleted
18	PHC workers should know the milestone of computer technology evolution	not valid (2.6)	deleted
19	PHC workers should be able to use World Wide Web (www) to search for information.	valid (4.4)	valid (4.7)
20	PHC workers should be able to receive and send an email and transfer file through the network	valid (4.9)	valid (4.7)
21	PHC workers should be able to use computerized self-learning device, such as e-learning, CD learning	valid (4.4)	valid (4.3)
22	PHC workers should know about the health information system, such as primary health care information system, hospital management information system	valid (4.3)	valid (4.8)
23	PHC workers should know the health information system in their workplace	valid (4.2)	valid (4.6)
24	PHC workers should know that the health information system is a tool for health service efficiency in their workplace	valid (4.4)	valid (4.7)
25	PHC workers should know the milestone of health information system evolution in their workplace	valid (3.4)	valid (4.4)
26	PHC workers should know about the network and computer application that have been used in the health information system in their workplace	valid (4.0)	valid (4.4)
27	PHC workers should know about the computer applications that can help them to make a decision	not valid (3.8)	deleted
28	PHC workers should know about the computer applications that can help them to perform their daily tasks (health environment, health promotion, etc.)	valid (4.2)	valid (4.2)
29	PHC workers should know about the computer device that can be used in medical treatment	not valid (3.3)	deleted
30	PHC workers should know how to use a computer for personal use	valid (4.4)	valid (4.4)
31	PHC workers should be able to use a word processing software to process documents for their daily tasks	valid (4.6)	valid (4.7)
32	PHC workers should be able use a spreadsheet program (e.g., Excel) to do simple data analysis	valid (4.7)	valid (4.7)
33	PHC workers should be able to use presentation editing software (Power Point) for presentation and education	valid (4.5)	valid (4.7)
34	PHC workers should be able to use database software to create a database that helps them to do their daily tasks.	valid (4.0)	valid (4.5)
35	PHC workers should be able to use the health information system to complete their work	valid (4.1)	valid (4.5)
36	PHC workers should be able to maintain a health information system that is used in their workspace	not valid (3.6)	deleted
37	PHC workers should be able to use the health information system to save, retrieve, and transfer data in their workplace	valid (4.2)	valid (4.7)
38	PHC workers should be able to use computer appliances that have been used in health service and medical service	valid (4.2)	valid (4.3)
39	PHC workers should be able to use software for making the website	not valid (3.4)	deleted
40	PHC workers should be able to make multimedia file for the website	not valid (3.5)	deleted
41	PHC workers should know how to use statistics software for research and for their daily tasks	not valid (3.8)	deleted
42	PHC workers should be able to use statistics software for research and fortheir daily tasks	valid (4.4)	valid (4.0)
43	PHC workers should know how to manage and save files	valid (4.4)	valid (4.7)
44	PHC workers should be able to convert a file to different application formats, such as Word to PDF	valid (4.0)	valid (4.3)
45	PHC workers should be able to use computer devices, such as printer, scanner	valid (4.4)	valid (4.6)
46	PHC workers should know how to make multimedia file for the website	not valid (3.5)	deleted
47	PHC workers should know how to edit multimedia file	not valid (3.4)	deleted
48	PHC workers should be able to use computerized self-learning device, such as e-learning, CD learning	valid (4.0)	valid (4.2)
49	PHC workers should know what is a computer program	not valid (3.3)	deleted
50	PHC workers should know what is algorithm	not valid (2.7)	deleted
51	PHC workers should know what is the characteristic of a good computer program	not valid (3.4)	deleted
52	PHC workers should be able to communicate with computer programmer	not valid (3.4)	deleted
53	PHC workers should know the procedure of making application for the health information system	not valid (3.4)	deleted
54	PHC workers should be able to design the flowchart of health information system	not valid (3.7)	deleted
55	PHC workers should be able to understand the flowchart of health information system	not valid (3.8)	deleted
56	PHC workers should know the importance of integration prodedure before designing computer program	not valid (3.3)	deleted
57	PHC workers should know that computer program has a limitation on design and capacity	not valid (3.6)	deleted
58	PHC workers should know that computer program is not intelligent and should be programmed based on the need	not valid (3.6)	deleted
59	PHC workers should know that the computer program is a tool for effectivities and efficiency and cannot replace the role of health professionals	valid (4.0)	PHC workers should know *that a computer program is a tool for effectivities and efficiency (4.0)*
60	PHC workers should know that the health information system has limitation and reliability	valid (4.0)	valid (4.5)
61	PHC workers should know the reason for a slow response of a computer program, such as many users at the same time	valid (3.6)	valid (4.4)
62	PHC workers should know that files in the computer are needed to back up	valid (4.4)	valid (4.5)
63	PHC workers should know the problems in data integration	not valid (3.7)	deleted
64	PHC workers should know that computer users usually do the mistakes	not valid (3.8)	deleted
65	PHC workers should know that computer programs recently do not have the ability to translate daily language	not valid (3.7)	deleted
66	PHC workers should know the importance of computer technology in daily tasks	not valid (3.9)	deleted
67	PHC workers should know that computers can be used as a tool for staffing and controlling	valid (4.0)	valid (4.3)
68	PHC workers should know that the computer can cause the dehumanization of patient care	not valid (3.7)	deleted
69	PHC workers concern how data have been collected and used.	valid (4.3)	valid (4.5)
70	PHC workers should know the importance of confidentiality when processing data in the medical record and in computer	valid (4.3)	valid (4.7)
71	PHC workers should know the regulation concerning about protecting personal information on the computer.	valid (4.4)	valid (4.4)
72	PHC workers should know the basic technic for encryption and control access, such as making a password to open a file (e.g., Word, Excel)	not valid (3.9)	deleted
73	PHC workers should know the copyright	not valid (3.9)	deleted
74	PHC workers should know what is the computer virus	not valid (3.8)	deleted
75	PHC workers should know how to prevent and to handle the computer virus	not valid (3.8)	deleted
76	PHC workers should know that computer needs to be learned so that it can be used as a tool for effectivity and efficiency	not valid (3.8)	deleted
77	PHC workers should know where they can find the resources to solve the computer problems	not valid (3.5)	deleted
78	PHC workers should know that the internet can be used as health information resources	valid (4.9)	valid (4.6)
79	PHC workers should know what kind of health information can be found on the internet.	valid (4.9)	valid (4.5)
80	PHC workers should know where they can find useful health information on the internet	valid (5.0)	valid (4.5)
81	PHC workers should know how to find useful health information on the internet	valid (5.0)	valid (4.7)
82	PHC workers should know how to use the internet to answer questions about health	valid (4.7)	valid (4.6)
83	PHC workers should be able to evaluate the health information that has been found on the internet	valid (4.4)	valid (4.5)
84	PHC workers should be able to tell the quality of health information found on the internet	valid (4.4)	valid (4.7)
85	PHC workers should know how to use health information that can help in their daily tasks	valid (4.5)	valid (4.5)
Added 1	–	–	*PHC workers should know the importance and the advantages of data to my work*.
Added 2	–	–	PHC workers should know *that a computer program cannot replace the role of the health professional*
	85	43	46

#### Round 1: Delphi panel

In the first round, PHIC4PHC ver.0 consisting of 85 items (Table 3) was distributed to 11 academicians. They were asked to judge the importance of each question using a Likert scale ranging from 1 (*strongly unimportant*) to 5 (*strongly important*). The expert judgments of academicians were crucial to improving the content validity of the questionnaire. Items were accepted in the questionnaires construction using a mean cutoff value of 4 with standard deviation (SD) ≤ 0.75 to gain a consensus (Hsu and Sandford, 2007). In this round, 42 items with a mean score of less than 4 were removed from the questionnaire construction. Furthermore, this process also eliminated the *security knowledge* indicator from the *ethical proficiency* domain. In this round, academicians reached a consensus. Table 2 shows the mean scores and ranking from the panel round. This round resulted in PHIC4PHC ver.1 (Table 3) which was later distributed to PHC experts.

#### Round 2: Delphi panel

In the second round, PHIC4PHC ver.1 was distributed to 13 PHC experts. The second Delphi panel added two items and reinstated one item that was judged to be unimportant by the academicians and withdrawn in the first round. These three items related to the importance of handling data and troubleshooting errors. This study labeled all items with a mean score ≥ 4 and SD ≥ 0.75 as important. The second round resulted in the PHI4PHC v.2 questionnaire (Table 3).

Table 2 shows that the academicians and PHC experts had different views about the importance of the main categories. Academicians judged the *health information literacy* category as the most important, whereas for the PHC expert, the *technical proficiency*, *ethical proficiency*, and *health information literacy categories* were the highest ranking.

### Stage 2: pretesting of the questionnaire

#### Pilot Study 1

The PHI4PHC v.2 questionnaire was distributed to 40 staff at the PHC in Gunung Pati, Semarang. Table 5 shows the characteristics of the respondents in the pilot study.

The PHI4PHC v.2 questionnaire comprised 46 items rated using a 5-point Likert scale ranging from 1 (*strongly disagree*) to 5 (*strongly agree*). The construction of the PHI4PHC v.2 questionnaire included both positive and negative statements to prevent any tendency in the respondents to give the same answers. Based on PHC experts’ advice, the questionnaire included three additional items asking about computer troubleshooting skills.

The results of validity and reliability testing indicated that two questions were not valid, with item-total correlation ≤ 0.263 and a Cronbach’s alpha coefficient of 0.956. The construction of the questionnaire resulting from the pilot study was discussed with the PHC experts, who still judged that two of the items were important for measuring PHIC. Based on the discussion, the sentence ‘I know the regulations concerning the protection of personal information on computer’ was revised to ‘I know the regulations concerning the protection of patient identity on computers.’ The sentence ‘I can determine the quality of health information found via the internet’ was revised to ‘I can differentiate between correct and incorrect information found via the internet.’ This step resulted in the PHIC4PHC v.3 questionnaire (Table 4).

**Table 4.** The Reducing Items Questionnaire on pretest

		Pretest	
No	Statements (PHIC4PHC ver.2)	PHIC4PHC v.3	Draft PHIC4PHC
1	I know recent computer systems, such as Windows, Macintosh.	valid (0.52)	not valid (0.24)
2	I know the basic usage of a computer, such as shutting down, using a mouse.	valid (0.27)	valid (0.28)
3	I know the file management function of the operating systems, such as creating, copying, moving folders or files.	valid (0.66)	valid (0.55)
4	I know how to use an operating system, such as Windows.	valid (0.52)	valid (0.55)
5	I do not know how to install drivers for the computer appliance, such as printers, scanner	valid (0.68)	valid (0.46)
6	I can solve common and simple computer error	valid (0.49)	valid (0.49)
7	I cannot use the World Wide Web (www) to search for information	valid (0.37)	valid (0.49)
8	I can receive and send emails to transfer files through the network	valid (0.62)	valid (0.46)
9	I cannot use computerized self-learning, such as e-learning, CD learning	valid (0.68)	valid (0.65)
10	I know about health information systems, such as primary health care information systems, hospital management information systems.	valid (0.59)	valid (0.55)
11	I know the health information system in the workplace	valid (0.31)	valid (0.64)
12	The health information system is a tool for health service efficiency in the workplace	valid (0.62)	valid (0.67)
13	I know the milestone in the evolution of health information system in the workplace	valid (0.74)	valid (0.62)
14	I do not know the network and computer application used in the health information system in the workplace	valid (0.31)	valid (0.69)
15	I know computer applications that can help me perform daily tasks.	valid (0.68)	valid (0.46)
16	I do not know how to use a computer for personal use	valid (0.43)	valid (0.54)
17	I can use word processing software to process documents for my daily tasks	valid (0.81)	valid (0.55)
18	I cannot use a spreadsheet program (e.g., Excel) to do simple data analysis	valid (0.68)	valid (0.44)
19	I cannot use presentation editing software (e.g., Power Point) for presentation and education	valid (0.84)	valid (0.45)
20	I can use database software to create a database for daily tasks.	valid (0.70)	valid (0.58)
21	I can use the health information system to complete my work	valid (0.56)	valid (0.49)
22	I can use the health information system to save, retrieve, and transfer data in the workplace	valid (0.73)	valid (0.69)
23	I cannot use computer appliances that used in the health service and medical service	valid (0.63)	valid (0.52)
24	I cannot use statistics software for research and daily tasks	valid (0.64)	valid (0.39)
25	I know how to manage and save files	valid (0.63)	valid (0.49)
26	I can convert a file to different application formats, such as Word to PDF	valid (0.82)	valid (0.62)
27	I cannot use computerized devices, such as printer, scanner	valid (0.53)	valid (0.64)
28	I cannot use computerized self-learning, such as e-learning, CD learning	valid (0.63)	valid (0.62)
29	Computer programs are tools for effectivities and efficiency	valid (0.53)	valid (0.52)
30	Computer programs can replace the role of health professionals	valid (0.75)	not valid (0.02)
31	Health information systems have limits to their reliability	valid (0.78)	not valid (0.20)
32	I do not know the reasons for the slow response of a computer program, such as many users at the same time	valid (0.41)	valid (0.47)
33	Files on the computer must be backed up	valid (0.68)	valid (0.50)
34	I know the importance and advantages of data to my work.	valid (0.72)	valid (0.48)
35	Computers can be used as a tool for staffing and controlling	valid (0.72)	valid (0.55)
36	I am concerned about how data have been collected and used.	valid (0.83)	valid (0.55)
37	I know the importance of confidentiality when processing data in the medical record and computers.	valid (0.72)	valid (0.58)
38	I know the regulation concerning the protection of personal information on the computer.	*not valid (0.23), changed to ‘I know the regulations concerning the protection of patient identity on computers’*	valid (0.49)
39	I know that the internet can be used as health information resources	valid (0.41)	valid (0.51)
40	I know what kind of health information can be found on the internet.	valid (0.54)	valid (0.43)
41	I know where to find useful health information on the internet	valid (0.51)	valid (0.42)
42	I know how to find useful health information on the internet	valid (0.41)	valid (0.59)
43	I know how to use the internet to answer questions about health	valid (0.50)	valid (0.64)
44	I can evaluate health information found on the internet	valid (0.47)	valid (0.56)
45	I can determine the quality of health information found on the internet	*not valid (0.10) change to ‘I can differentiate between correct and incorrect information found on the internet’*	valid (0.35)
46	I know how to use health information found on the internet to help in my daily tasks	valid (0.66)	valid (0.61)
	46	46	43

**Table 5.** Characteristics of the participants

	Delphi process		Pretest		Field test
Demographic characteristics	Expert 1 (N = 13) % (*n*)	Expert 2 (N = 11) % (*n*)	Pilot Study 1 (N = 40) % (*n*)	Pilot Study 2 (N = 35) % (*n*)	Validation study (N = 462) % (*n*)
Gender					
Male	53.8 (7)	36.4 (4)	20.0 (8)	28.6 (10)	15.4 (71)
Female	46.2 (6)	63.6 (7)	80.0 (32)	71.4 (25)	84.6 (391)
Education					
Senior high school	0.0 (0)	0.0 (0)	0.0 (0)	8.6 (3)	0.9 (4)
Vocational	61.5 (8)	0 (0)	65.0 (26)	51.4 (18)	84.6 (391)
Bachelor	30.8 (4)	0 (0)	32.5 (13)	40.0 (14)	14.1 (65)
Master	7.7 (1)	90.9 (10)	2.5 (1)	0.0 (0)	0.4 (2)
Doctoral	0 (0)	9.1 (1)	0.0 (0)	0.0 (0)	0.0 (0)

#### Pilot Study 2

This study distributed the PHIC4PHC v.3 questionnaire to 35 PHC workers at a monthly meeting at Semarang Municipality Health Office. In this pilot study, three questions had a total item correlation ≤ 0.283 (Items 1,30,31) and a Cronbach’s alpha coefficient of 0.923. The result of the discussion with the experts revealed items that could be withdrawn from the construction because the content could be represented by other items. In this pilot study, the experts reached a consensus on the construction of the questionnaire, thus signaling it the end of the process. This second pilot study generated the Draft PHIC4PHC. Table 4 shows the the questionnaire items.

### Stage 3: field testing the PHIC4PHC questionnaire

In this stage, the Draft PHIC4PHC questionnaire from Pilot Study 2 was validated to 462 PHC workers in Kendal District. The results of validity testing indicated that Item 13 (‘*I do not know network and computer application that have been used in the health information system in the workplace’)* was not valid with item-total correlation of 0.087, and therefore question was removed from the construct and the following process. The next reliability test for 42 items resulted in a Cronbach’s alpha coefficient of 0.946 for the entire scale ranging from 0.944 to 0.946. All of the items were valid with an item-total correlation ranging from 0.37 to 0.80 (Table 6).

**Table 6.** Items analysis of the final PHIC4PHC questionnaire

No	Questionnaire items/attributes	Mean	SD	r	Cronbach’s alpha if item deleted
1.1	*Health information system knowledge*				
1	I know about the health information system, such as primary health care information system, hospital management information system	3.75	0.963	0.68	0.944
2	I know about the health information system in my workplace	3.65	0.981	0.66	0.944
3	I know the milestone in the evolution of health information system in my workplace	2.91	1.111	0.68	0.946
4	I know about the computer applications that can help me to perform the daily tasks.	2.76	1.041	0.58	0.945
5	Computers can be used as a tool for workersing and controlling	2.85	0.982	0.6	0.946
6	I know the importance and the advantages of data to my work.	3.91	0.767	0.69	0.945
7	I am concern about how data have been collected and used.	3.82	0.76	0.73	0.945
8	A health information system is a tool for health service efficiency in my workplace	3.84	0.829	0.67	0.945
1.2	*Health information system skills*				
9	I can use health information systems to complete my work	2.98	1.06	0.61	0.946
10	I can use health information system to save, retrieve, and transfer data in the workplace	3.16	1.057	0.75	0.944
11	I cannot use computer appliances used in health service and medical service	3.24	0.979	0.67	0.945
2.1	*General computer skills*				
12	I can solve common and simple computer errors.	4.15	0.781	0.63	0.945
13	I do not know how to use the computer for personal use	3.11	1.027	0.67	0.944
14	I cannot use computerized device, such as printer, scanner	3.65	0.954	0.55	0.944
15	I do not know the reason for a slow response of computer program, such as many users at the same time	3.37	1.037	0.73	0.945
16	Computer programs are tools for effectiveness and efficiency	3.71	0.863	0.37	0.944
17	Files on a computer must be backed up	3.96	0.674	0.64	0.946
18	I know the basic usage of a computer, such as shutting down and using a mouse.	4.01	0.675	0.62	0.945
19	I know how to use an operating system, such as Windows.	4.10	0.711	0.76	0.945
20	I know how to manage and save files	4.11	0.648	0.63	0.945
21	I do not know how to install drivers for the computer devices, such as printers and scanners.	3.57	0.888	0.76	0.944
2.2	*Office application skills*				
22	I know the file management function of operating systems, such as create, copy, move folder or file	3.91	0.735	0.51	0.945
23	I can use word processing software to process documents for daily tasks.	3.67	1.04	0.66	0.946
24	I cannot use a spreadsheet program (e.g., Excel) to perform simple data analysis.	3.32	0.988	0.72	0.944
25	I cannot use presentation editing software (Power Point) for presentations and education	3.31	0.993	0.72	0.944
26	I cannot use statistics software for research and daily tasks.	2.98	1.005	0.64	0.945
27	I can use database software to create a database that is needed in my daily task.	3.45	1.081	0.45	0.944
28	I can use a computer as a self-learning tool	2.94	1.041	0.48	0.945
29	I cannot use computerized self-learning, such as e-learning, CD learning	3.58	0.892	0.76	0.945
30	I can convert a file to different application formats, such as Word to PDF	3.75	0.81	0.74	0.944
2.3	*Network skills*				
31	I cannot use the World Wide Web (www) to search information	3.21	1.073	0.79	0.945
32	I can receive and send emails to transfer files through the network	3.7	0.935	0.48	0.945
3	*Security and Legal Knowledge*				
33	I know the importance of confidentiality when processing data in the medical records and on computers	3.8	0.813	0.74	0.944
34	I know the regulations concerning the protection of patient identity on computers	3.76	0.827	0.77	0.944
4.1	*Health information access*				
35	I know what kind of health information can be found on the internet.	3.45	0.975	0.61	0.945
36	I know where to find useful health information on the internet	3.92	0.758	0.42	0.946
4.2	*Health information management*				
37	I know that the internet can be used as the health information resources	3.38	1.015	0.64	0.945
38	I know how to find useful health information on the internet	3.7	0.85	0.8	0.944
4.3	*Health information integration*				
39	I know how to use the internet to answer questions about health	3.35	0.994	0.66	0.944
40	I know how to use health information that had been found to help my daily tasks	3.24	1.073	0.76	0.945
4.4	*Health information evaluation*				
41	I can evaluate health information found on the internet	3.42	1.042	0.77	0.945
42	I can differentiate between correct and incorrect information found on the internet	3.22	1.042	0.68	0.944

### Data analysis

This study applied two-stage data processing. In the first stage, a statistical method was used to validate the PHIC4PHC questionnaire. Pearson’s product-moment correlation was used to determine the internal consistency; the Cronbach’s alpha coefficient was measured for each item as well as for the entire scale. The instrument has acceptable reliability if the Cronbach’s alpha coefficient is 0.70 or above. The statistical analyses were performed using the Statistical Package for Social Science 19 (Nie *et al*., 1975; Bryman and Cramer, 2012).

In the second stage, the results of the field test with 42 attributes were clustered into 3 categories using a data mining technique with K-means using tool Rapid Miner 8.1 (Kotu and Deshpande, 2014). Because the PHIC4PHC questionnaire is the first tool developed to measure PHIC in developing countries, no standard categories are available for judging PHIC in such cases. Therefore, this study applied K-means to categorize the PHIC standard into three unsupervised clusters. This study classified three clusters because previous studies have widely reported the results of analyzing data sets commonly containing three clusters of observations (Hill *et al*., 2006).

K-means is an unsupervised machine learning algorithm that clusters data, that are similar to one another into one cluster, which is then applied to unlabeled attribute. The K-means algorithm determines a set of K clusters and assigns each datum to exactly one cluster consisting of similar data. The similarity between data is based on a distance measure between them (Gan *et al*., 2007). The parameters set in the K-means algorithm were K (3), max run (10), measure type (Bregman divergence), max optimization steps (100), and divergence (square Euclidean distance).

**Figure 2.**
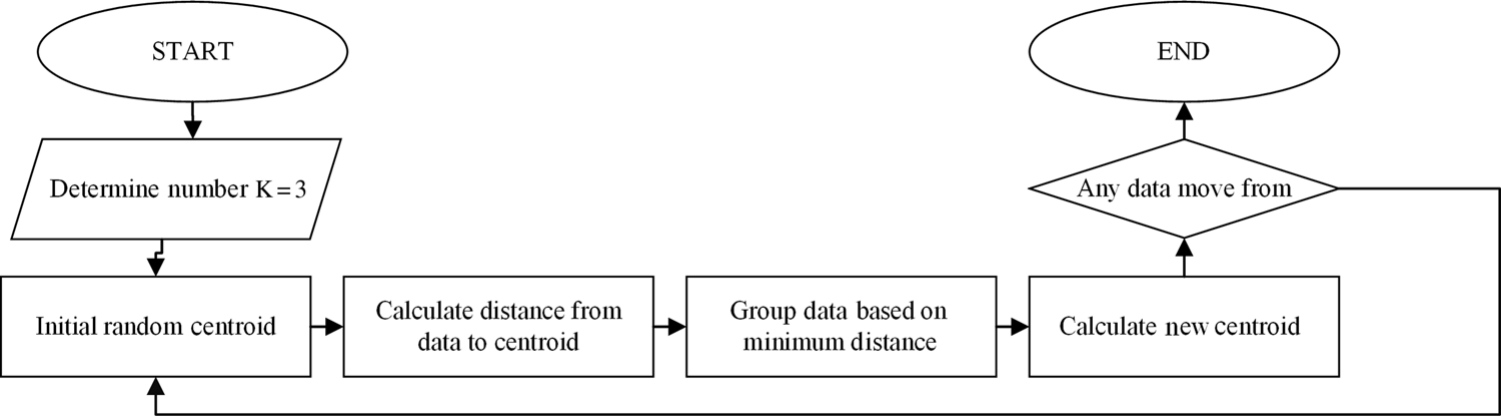
The K-means algorithm

Figure 2 shows K-means algorithm categorization process:

1. Determine the K value (3) as the number of categories and the metric dissimilarity (distance).
2. Randomize the initial centroid of each category that will be used to the cluster data.
3. Allocate all data to the nearest centroid by calculating the distance from the data to the centroid. This study used Euclidean distance to find the distance, as follows:
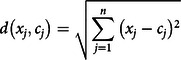

4. Recalculate centroid C based on data that follows each cluster as follows:
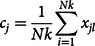
where Nk is the amount of data that incorporated in a cluster.
5. Repeat Steps 3 and 4 until convergence is reached when no data switch clusters (Shidik *et al*., 2016).

## Result

### Participants of the study

#### Delphi panel

This study recruited two groups of experts to test the initial questionnaire developed based on the literature review. The criterion for expert selection was experiences and knowledge of PHC. This stage involved 11 academicians from the Faculty of Health Science, Dian Nuswantoro University, Semarang and 13 public health practitioners from Semarang Municipality Health Office. Their average age was 40.5 years and the average work experience was 10.3 years. Table 5 shows the experts profiles.

#### Pretest and field test

Two pilot studies were conducted for pretest study followed by a field test. In the two pilot studies, the questionnaire was distributed to 40 and 35 PHC workers on Semarang District; in the field test, questionnaire was distributed to 462 PHC workers in Kendal District, Indonesia.

Table 5 shows the characteristics of all of the participants in this study. The proportion of women was higher than that of men among the PHC workers. The most common educational background was vocational school, particulary in PHC.

### Validating PHIC4PHC

PHIC4PHC was developed through the standard process of questionnaire development, consisting of the construction of items based on the literature and expert judgment, a pilot test and field test (Boynton and Greenhalgh, 2004; Rattray and Jones, 2007). The validation process is shown in Tables 3 and 4. The final PHIC4PHC questionnaire had 42 questions, as shown in Table 6.

The final results of PHIC4PHC were then clustered into three categories, based on the K-means algorithm. The results had normal distribution with 45.7% achieveing medium competency, 25.6% achieveing low competency, and 27.7% achieving high competency.

Table 7 shows that the highest proportion of PHC workers was in the medium category of PHIC (45.7%). Table 8 shows the category distribution for the level of competencies among the PHC workers.

**Table 7.** Centroid attributes of PHIC4PHC cluster using K-means

Attribute	Low PHIC	Medium PHIC	High PHIC
1	3.7	4.1	4.6
2	2.8	3.9	4.5
3	2.8	3.7	4.4
4	2.7	2.5	3.8
5	2.2	2.7	3.5
6	3.2	3.5	4.4
7	2.4	3.5	4.4
8	2.6	2.7	3.8
9	2.2	3.2	3.9
10	2.6	3.6	4.0
11	3.6	4.0	4.2
12	2.7	3.5	3.9
13	2.4	3.5	4.0
14	2.9	3.2	4.3
15	2.3	3.3	4.0
16	2.8	2.9	4.2
17	2.6	2.9	4.2
18	2.3	3.2	3.9
19	2.4	3.5	3.9
20	2.3	3.5	4.0
21	2.8	3.0	4.0
22	2.6	2.7	3.8
23	2.8	3.7	4.4
24	2.3	2.8	3.8
25	2.9	3.1	4.2
26	3.0	3.8	4.3
27	3.7	3.9	4.3
28	2.6	2.6	3.6
29	3.5	3.9	4.4
30	3.5	3.8	4.2
31	3.7	4.0	4.3
32	3.5	3.9	4.3
33	3.7	4.0	4.5
34	3.1	3.7	4.2
35	3.7	4.1	4.5
36	3.2	4.0	4.2
37	3.1	3.9	4.2
38	3.0	3.9	4.2
39	2.9	3.9	4.2
40	2.8	3.7	4.0
41	2.9	3.7	4.0
42	3.0	3.9	4.2
Frequency of cluster	123	211	128
Percentage of cluster	26.6	45.7	27.7

**Table 8.** Category distribution of PHIC4PHC among PHC workers

Attribute	Category	Mean	Total		Low PHIC		Medium PHIC		High PHIC	
			f	%	f	%	f	%	f	%
Gender	Male	152.1	71	15.4	13	18.3	33	46.5	25	35.2
	Female	146.8	391	84.6	110	28.1	178	45.5	103	26.3
Age	<31 years	153.8	69	14.9	10	14.5	35	50.7	24	34.8
	31–40 years	149.4	197	42.6	48	24.4	91	46.2	58	29.4
	41–50 years	143.7	153	33.1	51	33.3	66	43.1	36	23.5
	>50 years	143.8	43	9.3	14	32.6	19	44.2	10	23.3
Education	High School	125.0	4	9	2	50.0	1	25.0	1	25.0
	Diploma	146.8	391	84.6	108	27.6	181	46.3	102	26.1
	Bachelor	152.8	65	14.1	13	20.0	29	44.6	23	35.4
	Master	184.5	2	4	0	0.0	2	100.0	0	0.0
Work experience	≤10 years	151.2	212	45.9	46	21.7	95	44.8	71	35.5
	11–20 years	148.4	118	25.5	31	26.3	56	47.5	31	26.3
	>20 years	141.6	132	28.6	46	34.8	60	45.5	26	19.7

Table 8 shows that men had higher PHIC than women, and the higher the level of education among the PHC workers, the higher their PHIC; the longer the work experience among the PHC workers, the lower their PHIC; and the older the PHC workers, the lower their PHIC4PHC score.

## Discussion

This PHIC4PHC is the first comprehensive questionnaire to assess the competencies required in the digital health era for PHC workers such as computer skills, ethical skills, and health literacy skills. Health literacy has become vital in the digital era because health professionals need to harness the myriad information sources as a consequence of the implementation of ICT in health care facilities (Jackson, 2014).

The implementation of ICT in health care institutions, particularly in low- to middle-income countries such as Indonesia, still raises the concerns about confidentiality and privacy (Koo *et al*., 2001; Luna *et al*., 2014). Accordingly, this study included confidentiality and privacy as one a measurement indicator to comprehensively capture PHIC.

In the first stage, this study used a modified Delphi technique, a popular strategy that combines quantitative and qualitative method (Murphy *et al*., 1989; De Villiers *et al*., 2005; Fong *et al*., 2013; Keller and Heiko, 2014). This study used the Delphi technique to gather the opinions and perspectives of experts, educator, and practitioners about the construction of the questionnaire because no measurement tools are available to measure PHIC in PHC, particularly in developing countries.

The round of the Delphi process that focused on the education experts resulted in the removal of the *security* indicator from the *ethical* domain in the initial construction. This indicator was related to knowledge about computer viruses and how to handle them. The ranking of the importance of indicators differed between the two groups of experts. The education experts ranked the *information* domain as the most important, whereas, for PHC experts, the importance was equal among domains. This study identified 42 items regarding PHIC that are crucial for PHC workers.

Unlike in previous studies, the results of PHIC4PHC were processed using a data mining technique because it provides the ability to detect the optimal combination of precise parameter that should be assigned to each of the variables for classification according to the purpose of this study (Tufféry, 2011). This study applied cluster analysis because this method is mostly used when no a priori hypotheses are available and research remains in the exploratory phase. Cluster analysis is an exploratory data analysis tool that aims at sorting different objects into groups such that the degree of association between two objects is maximal if they belong to the same group otherwise minimal. Furthermore, this study did not assess statistical significance among clusters because, unlike many other statistical procedures, cluster analysis is a ‘collection’ of different algorithms that ‘place objects into clusters’ according to similarity rules. Hence, statistical significance testing is not appropriate in cluster analysis (Hill *et al*., 2006).

PHIC are crucial for Indonesia’s public health workers because the specific geography of the thousands of islands of Indonesia pose a challenge for PHC service, particularly in rural areas of the country. ICT is a solution for improving effectiveness and efficiency in PHC service with the implication that public health professionals should be the earliest adopters of computers and other information technologies. PHIC will generate innovative ways to promote public health using information science and technology (Yasnoff *et al*., 2000).

PHIC4PHC revealed that women likely have lower PHIC than men in PHC, which is consistent with the results of previous studies that a gender issue remains in ICT implementation, particularly in developing countries, despite continual claims that IT is gender-neutral (Hafkin and Taggart, 2001; Hafkin and Huyer, 2007; Flynn-Dapaah and Tareq Rashid, 2010). The implementation of a HIS in PHC should contemplate gender issues at the early stages of ICT adoption to allow women to participate fully in using the HIS, particularly in the rural areas.

This study showed that the longer the work experience of public health practitioners, the lower their PHIC. This finding differs from that of previous studies because ICT literacy requires a certain amount of experience (Usluel, 2007). However, the result is unsurprising because although the duration of using ICT was related to ICT literacy, work experience was related to the age of public health practitioners and older public health practitioners have longer work experience. Older people generally have lower ICT competencies than younger people (Tijdens and Steijn, 2005), which is consistent with the finding of this study that the older public health practitioners had lower competency.

PHIC4PHC could fill the gap in the tools available for measuring the readiness of human resources in PHC institutions to adopt HISs because it can evaluate the PHIC of public health workers. The evaluation results can determine the work necessary to promote the competency of PHC workers in ICT, such as training for existing health workers and developing a curriculum, gender-specific training, education and work experience, and so forth (Hagdrup *et al*., 1999).

## Conclusion

This paper describes the research method for measuring PHIC in the form of a questionnaire comprising 7 indicators and 42 items. The primary indicators were cognitive proficiency, technical proficiency, ethical proficiency, and health information literacy.

Previous studies have measured PHIC in developed countries and typically for PHC workers in higher education. This PHIC4PHC is valid and reliable in measuring PHIC in urban and rural PHC facilities. The final version of the assessment tool developed in this study is expected to be used in the future study of PHI in PHC, particularly in developing countries and resource-limited settings to elevate the success of implementing ICT in health care service.
